# Characterization of *FLOWERING LOCUS C 5* in *Brassica rapa* L.

**DOI:** 10.1007/s11032-023-01405-0

**Published:** 2023-07-19

**Authors:** Ayasha Akter, Tomohiro Kakizaki, Etsuko Itabashi, Kohei Kunita, Motoki Shimizu, Mst. Arjina Akter, Hasan Mehraj, Keiichi Okazaki, Elizabeth S. Dennis, Ryo Fujimoto

**Affiliations:** 1 Graduate School of Agricultural Science, Kobe University, Rokkodai, Nada-ku, Kobe, 657-8501 Japan; 2 Department of Horticulture, Bangladesh Agricultural University, Mymensingh, 2202 Bangladesh; 3 Institute of Vegetable and Floriculture Science, NARO, Kusawa, Ano, Tsu, Mie 514-2392 Japan; 4 Iwate Biotechnology Research Center, Narita, Kitakami, Iwate, 024-0003 Japan; 5 Department of Plant Pathology, Bangladesh Agricultural University, Mymensingh, 2202 Bangladesh; 6 Graduate School of Science and Technology, Niigata University, Niigata, 950-2181 Japan; 7 CSIRO Agriculture and Food, ACT, Canberra, 2601 Australia; 8 Faculty of Science, School of Life Science, University of Technology Sydney, Broadway, Sydney, NSW 2007 Australia

**Keywords:** *FLOWERING LOCUS C*, Vernalization, Floral repressor, Transcription, *Brassica*

## Abstract

*Brassica rapa* L., which includes Chinese cabbage, turnip, and pak choi, has more complex flowering time regulation than does *Arabidopsis thaliana* due to the presence of multiple paralogous flowering time genes. *FLOWERING LOCUS C* (*FLC*) is one of the key genes regulating the flowering time, and *B. rapa* has four *FLC* paralogs. *BrFLC5* on the reference genome is deemed a pseudogene because of a mutation (from G to A) in the splice site of the third intron, but there are some accessions with a G nucleotide in the splice site. In this study, we genotyped 310 *B. rapa* accessions and found that 19 had homozygous and 81 had heterozygous putative functional *BrFLC5* alleles. Accessions of turnip showed the highest proportion with a functional *BrFLC5* allele. BrFLC5 acts as a floral repressor when overexpressed in *A. thaliana*. The *BrFLC5* expression level varied in pre-vernalized plants, and this transcriptional variation was not associated with the G/A polymorphism in the third intron. Three accessions having a higher *BrFLC5* expression in pre-vernalized plants had a 584-bp insertion in the promoter region. Many regions homologous to this 584-bp sequence are present in the *B. rapa* genome, and this 584-bp inserted region has tandem duplications of an AT-rich sequence in its central region. The possibility that a high expression of a functional *BrFLC5* could contribute to producing premature bolting-resistant lines in *B. rapa* vegetables is discussed.

**Supplementary Information:**

The online version contains supplementary material available at 10.1007/s11032-023-01405-0.

## Background

*Brassica rapa* L. comprises a large number of morphological variants and includes commercially important leafy vegetables such as Chinese cabbage (var. *pekinensis*), pak choi (var. *chinensis*), komatsuna (var. *perviridis*), root vegetables such as turnip (var. *rapa*), and oil seed/mustard (var. *trilocularis*, var. *dichotoma*, var. *oleifera*) (Lv et al. 2020). *B. rapa* leafy vegetables are grown mainly in Asian countries, and root vegetables are grown in European countries and the USA as well as in Asian countries. In India, oil seeds and mustard are the main crops (Qi et al. 2017). The whole-genome sequence of *B. rapa* was the first to be determined in the genus *Brassica* using the Chinese cabbage doubled haploid line, Chiifu-401-42 (Wang et al. 2011), followed by the whole genome sequence in yellow sarson (Belser et al. 2018) and pak choi (Li et al. 2020; Li et al. 2021). The reference genome sequence of Chiifu-401-42 was updated by long-read sequencing technology and high-through chromosome conformation capture (Hi-C) technology (Zhang et al. 2018).

Flowering time is one of the major influences on the quality of the harvested product and seed in the genus *Brassica*. *Brassica* species originally required prolonged cold exposure to induce flowering, and this phenomenon is called vernalization (Itabashi et al. 2018; Akter et al. 2018, 2021). *B. rapa* has two main flowering types; one requires little or no vernalization, and the other requires vernalization (Su et al. 2018). Even within *B. rapa* accessions requiring vernalization, there is a diversity in the degree of vernalization required, a variation of the length of the period of cold exposure for flowering. For oilseed or mustard, where seed is the product and crops are grown in warm climates, accessions with little or no vernalization requirement are used. Most leafy or root vegetables have a high vernalization requirement because early bolting leads to a decrease in quality of products, especially in spring Chinese cabbage that is sown in late winter or early spring (Akter et al. 2021).

The floral repressor, FLOWERING LOCUS C (FLC), a MADS-box transcription factor, mediates vernalization in *B. rapa* (Shea et al. 2019; Takada et al. 2019; Akter et al. 2021). Due to the whole genome triplication (WGT) in *B. rapa*, there are four copies of *BrFLC* (*BrFLC1*, *BrFLC2*, *BrFLC3*, and *BrFLC5*) (Shea et al. 2018; Akter et al. 2019, 2021). All four *BrFLC* paralogs are expressed in pre-vernalized plants and have active histone marks, histone H3 lysine 4 trimethylation (H3K4me3) and H3K36me3, in their coding regions (Mehraj et al. 2021). The expression of all four *BrFLC* paralogs gradually decreases during prolonged cold exposure (Akter et al. 2020). During this period, the repressive histone mark, H3K27me3, accumulates around the transcription start site of *BrFLC*s (Akter et al. 2019). After returning to a warm environment, H3K27me3 spreads across the entire encoding regions of all *BrFLC* paralogs, and the repression of their expression is maintained (Kawanabe et al. 2016; Akter et al. 2019, 2021).

There are variations in expression levels in pre-vernalized plants and in the degree of suppression of expression following vernalization among the four *BrFLC* paralogs in the same plant (Takada et al. 2019). Furthermore, there are variations in expression levels of each *BrFLC* gene between accessions in pre-vernalized plants and in the degree of suppression of expression following vernalization in each *BrFLC* gene between accessions (Takada et al. 2019). The total expression level of the four *BrFLC* paralogs in pre-vernalized and the degree of suppression of expression in each *BrFLC* following prolonged cold treatment are important factors for the intraspecific variation of the vernalization requirement (Takada et al. 2019; Akter et al. 2021). These two characteristics also contribute to the difference in vernalization requirements in canola (*Brassica napus*); winter types, which have high vernalization requirements, showed a high level of total *BnFLC* expression or residual *BnFLC* expression level in specific paralogs following prolonged cold treatment (Calderwood et al. 2021). Since the total *BrFLC* expression level in pre-vernalized plants is important, the loss of function of one *BrFLC* paralog will result in a reduction in the total expression level of functional *BrFLC*s and a reduction in the vernalization requirement. For example, the early flowering accession, yellow sarson, has a loss of function of *BrFLC1* and *BrFLC2* (Li et al. 2009; Yuan et al. 2009). Similarly, if one *BrFLC* paralog is not reduced in expression following prolonged cold treatment, the repression rate of total *BrFLC* expression will be reduced, resulting in a higher vernalization requirement. For example, in Tsukena No. 2, the expression levels of *BrFLC2* and *BrFLC3* are less reduced following prolonged cold treatment (Kitamoto et al. 2014), and the expression level of *BrFLC1* is less reduced in BRA2209 (Takada et al. 2019); these two accessions both have a high vernalization requirement.

*BrFLC5* is classified as a pseudogene due to the deletion of two exons in the reference genome (Schranz et al. 2002; Wang et al. 2011). Later, a polymorphism in the sequence of the splicing donor site of the third intron (G/A) was found among *B. rapa* accessions, and the presence of accessions encoding the complete *BrFLC5* gene was reported (Xi et al. 2018). Quantitative trait loci (QTLs) affecting flowering time in different populations of *B. rapa* have been identified (Shea et al. 2018; Akter et al. 2021), and some QTLs cover regions including *BrFLC1*, *BrFLC2*, or *VERNALIZATION INSENSITIVE3.1* (*BrVIN3.1*) (Zhao et al. 2010; Su et al. 2018). A flowering time QTL covering *BrFLC5* was identified (Kakizaki et al. 2011), and the possibility that *BrFLC5* is involved in flowering time variation has been shown (Kakizaki et al. 2011; Xi et al. 2018).

An accession encoding a complete *BrFLC5* has been identified, and there is a flowering time difference between plants having a functional (G) and non-functional (A) polymorphism at the 5′ splice site in the third intron of *BrFLC5* in the F_2_ population, suggesting that BrFLC5 contributes to the flowering time difference (Xi et al. 2018). Here, we identified accessions having a functional (G) or non-functional (A) polymorphism at the 5′ splice site in the third intron of *BrFLC5* from over 300 *B. rapa* accessions. We confirmed that BrFLC5 acts as a floral repressor by overexpressing *BrFLC5* in *Arabidopsis thaliana*. The variation of expression levels of *BrFLC5* in pre-vernalized plants among *B. rapa* accessions and the variation in the repression rate of *BrFLC5* expression following prolonged cold treatment were examined. Finally, we discuss whether *BrFLC5* can be used as a breeding resource.

## Materials and methods

### Plant materials and growth conditions

Three hundred ten *B. rapa* accessions from four different varieties were used to examine a polymorphism in the sequence of the splicing donor site of the third intron (G/A) (Kawamura et al. 2016) (Table S1). Ten *B. rapa* accessions (Chinese cabbage; Chukanbohon Nou 6 gou (Nou 6) (Kakizaki et al. 2011), “Eishun,” “Nozaki Hakusai No. 2” (Nozaki Saishujo Ltd., Japan), “CR Kisaku-80,” “Raiou-90” (Marutane Co., Ltd., Japan), “CR-Okiniiri,” “Homarenokiwami,” “Shoshun” (Takii & Co., Ltd., Japan), Turnip; “Aishinku No. 3,” “CR Takamaru” (Musashino Seed Co., Ltd., Japan)) were used to examine the expression of *BrFLC5* in pre-vernalized condition. After surface sterilization, the seeds were sown in agar-solidified Murashige and Skoog (MS) plates with 1% (w/v) sucrose under long-day (LD) conditions (16-h light) at 22 °C. Fourteen-day seedlings on MS plate were treated for 4 weeks at 4 °C under LD conditions. F_2_ seeds were produced by self-pollinating by bud pollination of F_1_ crossed by Nou 6 and “Eishun.” F_2_ seeds were sown in Petri dishes containing moistened paper and then transplanted into cell trays filled with soil. At 14 days after sowing, one leaf was used for RNA extraction and the remaining leaves for DNA extraction.

### Sequencing DNA fragments of BrFLC5 gene

Genomic DNAs were isolated from 14-day first and second leaves by the cetyl trimethyl ammonium bromide (CTAB) method (Murray and Thompson 1980). The regions including the third intron were amplified by PCR using a primer set, BrFLC5-F/R (Fig. S1; Table S2). Quick Taq® HS DyeMix (Toyobo Co., Ltd., Osaka, Japan) was used for PCR. Amplified PCR products were treated by illustra ExoProStar (GE Healthcare Life Sciences, Chicago, Illinois, USA) and were directly sequenced using ABI Prism 3130 (Applied Biosystems, Foster City, California, USA).

The 1355- or 771-bp upstream regions from the transcription start site (TSS) were amplified by PCR using a primer set, BrFLC5_pro-F1/R1 (Fig. S1; Table S2). Amplified PCR fragments (771 bp) of seven accessions (“Nozaki Hakusai No. 2,” “CR Kisaku-80,” “Raiou-90,” “Eishun,” “Shoshun”, “Homarenokiwami,” and “CR-Okiniiri”) were directly sequenced. Amplified PCR products (1355 bp) of three accessions (Nou 6, “Aishinku No. 3,” and “CR Takamaru”) were cloned into pGEM®-T Easy vector (Promega, Madison, WI, USA). Quick Taq® HS DyeMix (Toyobo) was used for PCR. Nucleotide sequences of five clones of PCR products were determined with the ABI Prism 3130 (Applied Biosystems). Primers, M13F/R and BrFLC5_pro F2/R2, were used for sequencing (Fig. S1; Table S2). The data were analyzed using Sequencher (Gene Codes Corporation, MI, USA). Genotyping of F_2_ plants derived from Nou 6 and “Eishun” was performed by PCR using a primer set, BrFLC5_pro-F1/R1 (Fig. S1; Table S2). PCR was performed using the following conditions: 1 cycle of 94 °C for 2 min, 35 cycles of 94 °C for 30 s, 58 °C for 30 s, and 68 °C for 2 min. Primer sequences and their position used for sequencing are shown in Fig. S1 and Table S2.

### RNA extraction and RT-qPCR

Total RNA was isolated from 14-day first and second leaves or from first and second leaves following 4 weeks of cold treatments to 14-day seedling using the SV Total RNA Isolation System (Promega). The leaves from three individual plants in each condition were harvested as biological replicates. The cDNA was synthesized from 500 ng total RNA using ReverTra Ace qPCR RT Master Mix with gDNA Remover (Toyobo). RT-qPCR was performed using LightCycler 96 (Roche Diagnostics International Ltd., Switzerland). The cDNA was amplified using FastStart Essential DNA Green Master (Roche). PCR conditions were 95 °C for 10 min followed by 40 cycles of 95 °C for 10 s, 60 °C for 10 s, and 72 °C for 15 s, and the melting program (60 °C to 95 °C at 0.1 °C/s). After amplification cycles, each reaction was subjected to melt temperature analysis to confirm single amplified products. The expression level of each gene relative to *BrACTIN* was automatically calculated using automatic CQ calling according to the manufacturer’s instructions (Roche) (Fujimoto et al. 2006). Data presented are the average and standard error (s.e.) calculated from three biological and experimental replications. Primer sequences used for RT-qPCR are shown in Table S2.

### RNA-sequencing

For RNA-sequencing (RNA-seq), total RNA from 14-day first and second leaves of pre-vernalized in “Aishinku No. 3,” “CR Takamaru,” Nou 6, and “Homarenokiwami” were used. A library was prepared using NEBNext® Ultra™ II Directional RNA Library Prep Kit for Illumina® (New England Biolabs, Ipswich, MA, USA), and sequencing was performed using NovaSeq 6000 (Illumina Inc., San Diego, CA) (paired-end, 150 bp). Low-quality reads were filtered using FaQCs ver 2.10 (Lo and Chain 2014), and HISAT2 ver 2.2.1 (Kim et al. 2015) was used to align the filtered reads to *Brassica rapa* reference sequence v1.5 (http://brassicadb.org/cn/). The levels of gene expression were scored by fragments per kilo-base per million (FPKM) using cuffdiff v2.2.1 (Trapnell et al. 2010). The RNA-seq data were deposited in the DNA Data Bank of Japan (DDBJ; accession no. DRA015525)

### Constructs and plant transformation

The cDNA from leaves of Nou 6 was used for RT-PCR. PrimeSTAR GXL DNA Polymerase (Takara Bio, Shiga, Japan) was used for RT-PCR. PCR fragments of a coding sequence (CDS) of *BrFLC5* were amplified by RT-PCR using a primer pair, BrFLC5_5UTR_Sal and BrFLC5_3UTR_XbaI-2 (Table S2). PCR was performed using the following conditions: 1 cycle of 94 °C for 2 min, 35 cycles of 94 °C for 30 s, 57 °C for 30 s, and 72 °C for 30 s. Amplified PCR products were then cloned into the pGEM®-T Easy vector (Promega). The *Sal* I and *Xba* I fragment including *BrFLC5* CDS was inserted between the 35S promoter and Nos terminator in the overexpression vector prepared by modifying the pCAMBIA1301 binary vector (Itabashi et al. 2019). Ligation was carried out using In-Fusion HD Cloning Kit (Takara Bio), and a primer pair, BrFLC5_CDS-fusF3 and BrFLC5_CDS-fusR3, was used (Table S2). This construct was transformed into *Agrobacterium tumefaciens* strain EHA105, and the transformation of Columbia-0 (Col) accession in *A. thaliana* was carried out by the floral dip procedure (Clough and Bent 1998). Transgenic seedlings were selected, which showed hygromycin resistance on a selection medium. T_2_ plant seeds were sown on MS medium and grown under LD conditions (16-h light) at 22 °C. After growing plants on MS medium, they were transferred to soil and grown under the conditions described above. The flowering time observed in *A. thaliana* was expressed as the number of rosette leaves at the time of flowering. Total RNA was isolated from mature leaves of T_2_ plants with and without transgene, and RT-PCR was performed to confirm the expression of the transformed *BrFLC5* gene. PCR conditions were 1 cycle of 94 °C for 2 min, 30 cycles of 94 °C for 30 s, 55 °C for 30 s, and 68 °C for 30 s. Primer sequences used for RT-PCR are shown in Table S2.

## Results

### Identification of functional and non-functional alleles in *B. rapa* germplasm

In the reference genome sequence of *B. rapa*, Chiifu-401-42, *BrFLC5* (Bra022771/BraA03g015950.3C) is annotated with five exons, while the other three *BrFLC* paralogs (*BrFLC1*, *BrFLC*2, and *BrFLC*3) are annotated with seven exons (Wang et al. 2011; Zhang et al. 2022), implying that *BrFLC5* is a pseudogene in *B. rapa*. The reason for the decrease in the number of exons is the presence of a mutation from G to A in the splicing donor site (GT) in the third intron (Fig. S1). However, some lines of *B. rapa* have G nucleotide in the splicing donor site; in these accessions, there are seven exons in *BrFLC5* (Xi et al. 2018). In this study, we examined the G/A polymorphism in 310 accessions of *B. rapa* that were mainly collected from Japanese commercial cultivars. Direct sequencing of the 673 bp sequence harboring this G/A polymorphism was performed in 310 *B. rapa* accessions (Fig. S1). Among the 310 accessions, 19 accessions (6.1%) had a homozygous G/G allele, 81 accessions (26.1%) had a heterozygous G/A allele, and 210 accessions (67.7%) had a homozygous A/A allele (Table 1). Among four varieties of *B. rapa*, the frequency of homozygous G/G alleles was 31.3% in turnip, 5.0% in komatsuna, 4.8% in Chinese cabbage, and none in pak choi. In all varieties, the frequency of a homozygous G/G allele was lower than that of a homozygous A/A allele (Table 1).

**Table 1:** Frequency of G/A polymorphism in the splicing donor site of the third intron of *BrFLC5* in *B. rapa* vegetables

		G/G		G/A		A/A		Total
		Number	%	Number	%	Number	%	Number
Chinese cabbage	var. *pekinensis*	11	4.8%	67	29.4%	150	65.8%	228
Turnip	var. *rapa*	5	31.3%	2	12.5%	9	56.3%	16
Komatsuna	var. *perviridis*	2	5.0%	11	27.5%	27	67.5%	40
Pak choi	var. *chinensis*	0	0.0%	0	0.0%	17	100.0%	17
Others		1	11.1%	1	11.1%	7	77.8%	9
Total		19	6.1%	81	26.1%	210	67.7%	310

The reason for the different frequency of a functional *BrFLC5* allele between Xi et al. (2018) and our study is that our plant materials included a higher percentage of Chinese cabbage accessions, and Xi et al. (2018) included more turnip and pak choi accessions. The frequency of homozygous G/G alleles differed between the two plant material sets, and the plant materials of Xi et al. (2018) contained a higher frequency of homozygous G/G alleles than ours. Turnip accessions had the highest percentage of homozygous G/G alleles in both plant materials (Fig. S2).

### BrFLC5 functions as a floral repressor

**Fig. 1:**
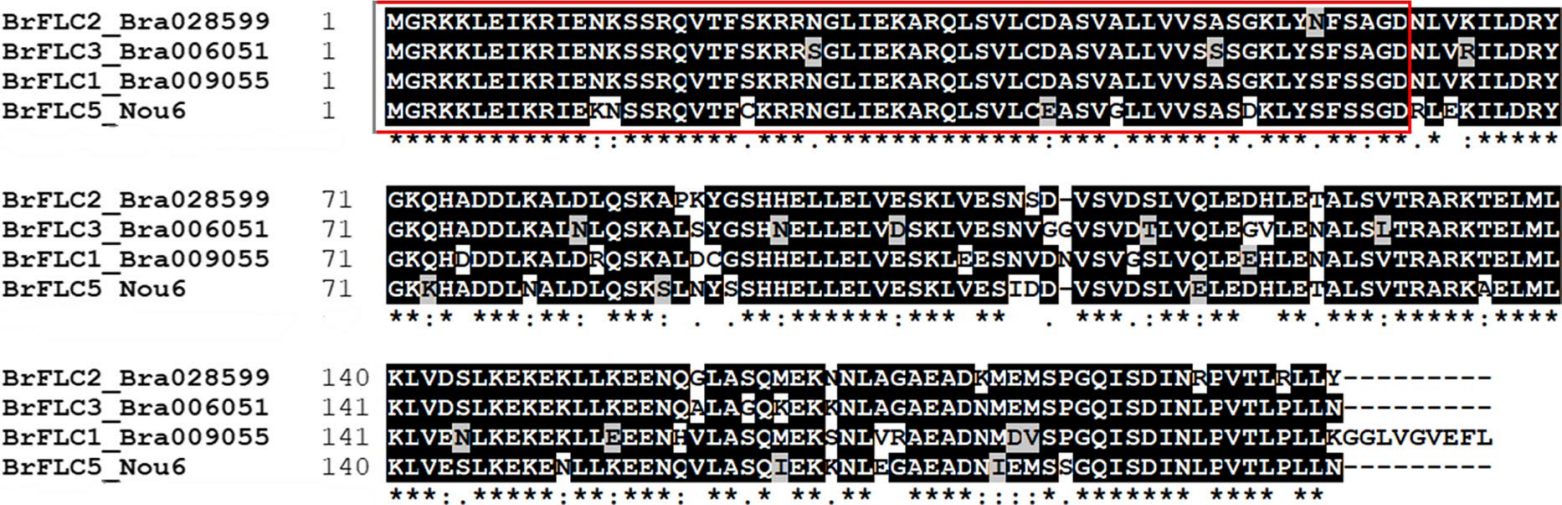
Comparison of the predicted amino acid sequences between BrFLC5 and other BrFLCs (BrFLC1, BrFLC2, and BrFLC3). The MADS-box domains are represented in a red box

BrFLC1, BrFLC2, and BrFLC3 but not BrFLC5 have been shown to act as floral repressors (Kim et al. 2007; Takada et al. 2019). The nucleotide sequence of the coding region of *BrFLC5* in Nou 6 was determined. The similarities of the predicted amino acid sequence of BrFLC5 to BrFLC1, BrFLC2, and BrFLC3 were 81%, 84%, and 80%, respectively (Fig. 1). The MADS-box domain responsible for binding to target DNA was conserved in BrFLC5 (Fig. 1).

**Fig. 2:**
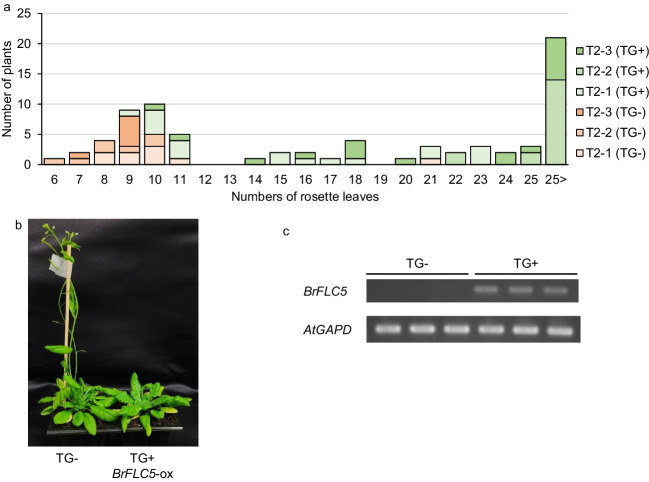
Overexpression of *BrFLC5* causes late flowering. **a** Number of rosette leaves in three T_2_ populations that are overexpressing *BrFLC5*. **b** Flowering time phenotypes in T_2_ plants with overexpressing *BrFLC5*. No cold (4 °C) treatments were applied, and the plants were grown under long-day conditions (16-h light) at 22 °C. **c** RT-PCR analysis showing transcription of *BrFLC5*. Three independent plants from the T_2_-2 population were used. *AtGAPD* was used as a control of demonstrate equal concentration of cDNA templates. TG+ and TG− represent the presence and absence of transgenes (TG), respectively

A 35S promoter::*BrFLC5* CDS was transformed into the Columbia-0 accession of *A. thaliana* that has no *AtFLC* expression because of the loss of function of *FRIGIDA* (*AtFRI*) (Johanson et al. 2000). More than twenty T_1_ plants were obtained. Some T_1_ plants did not flower and offspring were not obtained. Three T_2_ populations (T_2_-1, 2, and 3 populations) were examined for flowering time, and the flowering time of plants transgenic for *BrFLC5* transgene (TG+) was later than plants without the transgene (TG−) (Fig. 2a, b). All plants transformed with transgene did not show delayed flowering. This was also observed in previous reports (Kim et al. 2007; Itabashi et al. 2019; Takada et al. 2019), and Kim et al. (2007) suggested that the protein level of FLC might be the cause. The flowering time of plants transgenic for *BrFLC5* transgene (TG+) was also later than plants without the transgene (TG−) in two T_3_ populations; T_3_-1 and T_3_-2 populations were derived from T_2_-2 and T_2_-3 late flowering plants, respectively (Fig. S3). The expression of *BrFLC5* in transgenic plants was confirmed by RT-PCR (Fig. 2c).

### Expression levels of *BrFLC5* varied among *B. rapa* accessions

**Fig. 3:**
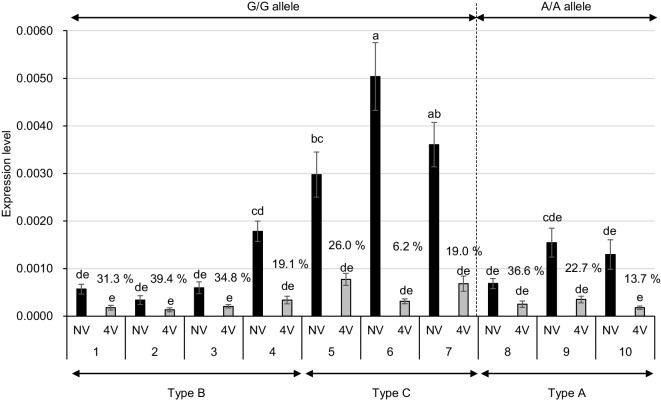
The expression levels of *BrFLC5* with (4V) and without (NV) 4 weeks of cold treatments. The expression level of *BrFLC5* relative to *BrACTIN* was calculated. Data presented are average and standard error (s.e.) of three biological and experimental replications. Percentages (%) shown above the bars are the ratio of expression level in 4V samples compared with NV samples. NV, pre-vernalized; 4V, 4 weeks of vernalized; G, functional allele; A, non-functional allele. The letters above the bars represent significant differences at *p*<0.05 (Tukey-Kramer test). Type A has no insertion in the promoter region and has an A/A homozygous allele. Type B has no insertion in the promoter region and has a G/G homozygous allele. Type C has a 584-bp insertion in the promoter region and has a G/G homozygous allele. 1, “Nozaki Hakusai No. 2”; 2, “CR Kisaku-80”; 3, “Eishun”; 4, “Raiou-90”; 5, Nou 6; 6, “Aishinku No. 3”; 7, “CR Takamaru”; 8, “Shoshun”; 9, “Homarenokiwami”; 10, “CR-Okiniiri”

We have suggested that the level of *FLC* expression prior to prolonged cold treatment and the rate of suppression of *FLC* expression following prolonged cold treatment are important in the vernalization requirement in *B. rapa* (Takada et al. 2019; Akter et al. 2021). The expression level of *BrFLC5* was examined by qPCR using a *BrFLC5*-specific primer set in ten accessions of *B. rapa* of which seven had homozygous G/G alleles and three had homozygous A/A alleles. “Aishinku No. 3” showed the highest expression levels followed by “CR Takamaru” and Nou 6, which all have a homozygous G/G allele (Fig. 3). The expression levels of “Homarenokiwami” and “CR-Okiniiri,” which have the homozygous A/A allele, were similar to that in “Raiou-90” and higher than those in “Nozaki Hakusai No. 2,” “CR Kisaku-80,” and “Eishun” that all have a homozygous G/G allele (Fig. 3), indicating that the expression level of *BrFLC5* in pre-vernalized plants was independent of the G/A polymorphism in its third intron.

**Fig. 4:**
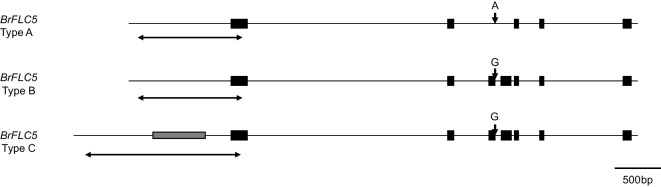
Two types of fragment sizes in the promoter region of *BrFLC5*. Type A has no insertion in the promoter region and has an A/A homozygous allele and includes three accessions (“Shoshun,” “Homarenokiwami,” “CR-Okiniiri”). Type B has no insertion in the promoter region and has a G/G homozygous allele including four accessions (“Nozaki Hakusai No. 2,” “CR Kisaku-80,” “Eishun,” “Raiou-90”). Type C has 584-bp insertion in the promoter region and a higher expression level of *BrFLC5* including in three accessions (Nou 6, “Aishinku No. 3,” “CR Takamaru”). Gray box represents the insertion and black boxes indicate the exons. G, putative functional allele; A, non-functional allele

The *BrFLC5* promoter sequences of ten accessions of *B. rapa* were determined. Among these ten accessions, there were two fragment sizes of promoter regions, 1335 bp (type C, G/G allele) and 771 bp (type A, A/A allele; type B, G/G allele) (Figs. 3 and 4). There were no sequence differences among accessions having the same fragment sizes, and the 771-bp sequence was identical to the sequences in the reference genome. The 1335-bp fragment has 584-bp insertion (Fig. 4), and all accessions having this insertion tended to have higher expression levels of *BrFLC5* before vernalization (Figs. 3 and 4). This 584-bp inserted region has an AT-rich sequence in its central region, and tandem duplications were found in this region (Fig. S4). Blast searches using this 584-bp sequence as a query revealed a homologous sequence in the promoter region of *FLC.A03b* of *B. napus* (Identity 583/584, 99.8%) that is an ortholog of *BrFLC5* (Akter et al. 2021); there were many regions homologous to this 584-bp sequence in the genome of *B. rapa* (Fig. S5).

An F_2_ population was produced by crossing Nou 6 (type C) and “Eishun” (type B), and eight F_2_ plants homozygous for the 584-bp insertion or without the 584-bp insertion were selected. The expression levels of *BrFLC5* of the eight F_2_ plants with a homozygous 584-bp insertion were higher than those without the 584-bp insertion (Fig. S6), suggesting that this 584-bp insertion is associated with a higher *BrFLC5* expression level.

In response to 4 weeks of cold treatment, *BrFLC5* expression levels decreased to between 6.2 and 39.4% of the non-vernalized level, and the average repression rate in accessions having G/G homozygous (25.1%) was similar to those having A/A homozygous (24.3%) (Fig. 3).

### *BrFLC5*showed the lowest expression level among four *BrFLC* paralogs

**Fig. 5:**
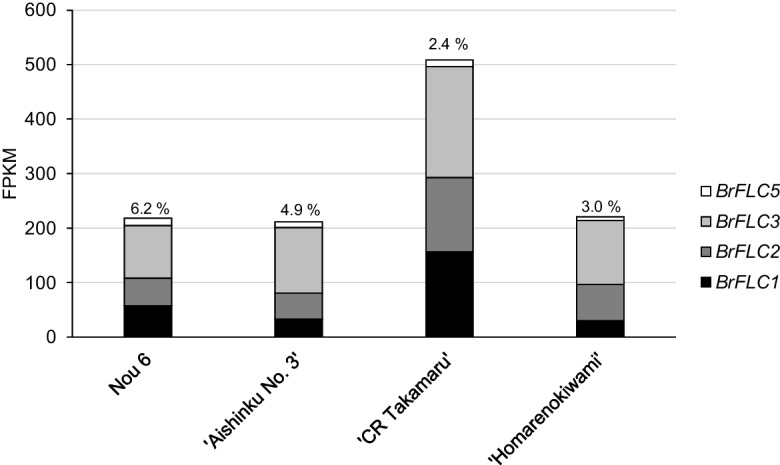
The expression level of *BrFLC1*, *BrFLC2*, *BrFLC3*, and *BrFLC5* genes in the four accessions of *B. rapa* from RNA-seq (FPKM value) data. FPKM, fragments per kilo-base per million. Percentages (%) shown above the bars are the ratio of expression level in *BrFLC5* in total *BrFLC* expression levels

We performed RNA-seq in the 14-day first and second leaves in four accessions of *B. rapa* (Nou 6, “Aishinku No. 3,” “CR Takamaru,” and “Homarenokiwami”); three of the four accessions (Nou 6, “Aishinku No. 3,” and “CR Takamaru”) have a homozygous G/G allele with 584-bp insertion in the *BrFLC5* promoter region. From 82.2 to 92.1% of total filtered reads (15.5- to 21.2-M reads) were mapped to the reference genome (Table S3). Expression levels of *BrFLC1*, *BrFLC2*, *BrFLC3*, and *BrFLC5* were identified using the FPKM value. *BrFLC3* had the highest expression levels in all four accessions, from 40.1 to 56.9% of the total *BrFLC* expression level, and *BrFLC5* had the lowest expression levels, from 2.4 to 6.2% of the total *BrFLC* expression level (Fig. 5).

## Discussion

Flowering time is an important trait for *B. rapa* vegetables, and FLC plays an important role in controlling flowering time in *B. rapa* (Akter et al. 2021). *BrFLC5* originated from the α replication of the common ancestor of *A. thaliana* and *B. rapa*, but it was not localized in the syntenic region where *AtFLC* is located (Yang et al. 2006; Akter et al. 2019). In the reference genome of *B. rapa*, *BrFLC5* was considered to be a pseudogene because it lacks two exons (Schranz et al. 2002; Wang et al. 2011). Furthermore, the expression level of *BrFLC5* is the lowest among the four paralogs in one accession (Xi et al. 2018), and in this study, similar results were found in four accessions. *BoFLC5* can also be a pseudogene and has the lowest expression levels among the paralogs in cabbage (*Brassica oleracea*) (Itabashi et al. 2019). The expression levels of *BnaFLC.A03b* and *BnaFLC.C03b*, which are orthologs of *BrFLC5* and *BoFLC5*, respectively, tended to be lower than the other *BnFLC* paralogs in *B. napus* (Calderwood et al. 2021). These results suggest that sequences that are associated with low expression levels of *FLC5* were fixed early after the speciation of *Brassica* species.

The contribution of functional *BrFLC5* to flowering time variation was shown using the F_2_ population derived from accessions having A homozygous and G homozygous alleles (Xi et al. 2018); BrFLC5 functions weakly to control flowering time even though other *BrFLC* paralogs affect the flowering time. QTL analysis using Nou 6 and A9709, which are high and low vernalization requirements, respectively, was performed in field conditions, sowing seeds in the winter season in Japan (January or February) without any cold treatment. Two QTLs were identified and one of them covered *BrFLC5* (Kakizaki et al. 2011). In this study, we confirmed that A9709 has a homozygous A allele, type A, and Nou 6 has a homozygous G allele, type C. Based on the data of Kakizaki et al. 2011 in four environmental conditions, the F_2_ plants having type C *BrFLC5* homozygous allele tended to have shorter stem length (higher vernalization requirement/later flowering time) than F_2_ plants having type A *BrFLC5* homozygous allele when *BrFLC1* allele was fixed (Fig. S7), suggesting that some of the flowering time difference of these two accessions might be due to the BrFLC5 function; differences between type A and type C of the *BrFLC5* allele were shown to be possibly related to differences in vernalization requirement (flowering time) (Kakizaki et al. 2011). In this study, we confirmed that BrFLC5 acts as a floral repressor. Integrating these results suggests that the functional difference of BrFLC5 (A allele, type A vs. G allele, type B/C) is associated with the difference in vernalization requirement/bolting time in *B. rapa*.

There are a few studies examining the relationship between *BrFLC5* and flowering time/vernalization requirement. However, some accessions having a full BrFLC5 amino acid sequence have been identified (Xi et al. 2018). We also identified some accessions with full annotation of the BrFLC5 amino acid sequence among 310 *B. rapa* accessions. Despite the differences in the populations used, the two studies showed that the frequency of the A allele (non-functional) is higher than the G allele (putative functional) and turnip showed the highest ratio of homozygous G allele. These results may be due to the stronger selection for vernalization requirements in turnip than in other *B. rapa* vegetables.

Transposable elements (TEs) have been found in the genomic regions in *FLC* in *B. rapa* and *B. napus* and sometimes affect the *FLC* expression levels before vernalization or the repression rate of *FLC* expression following prolonged cold treatments (Akter et al. 2021). We found a 584-bp insertion in the promoter region, and this sequence did not show any structures that would be considered TE but had tandem duplications. Accessions having this insertion tended to have higher *BrFLC5* expression levels than accessions without this insertion in pre-vernalized plants, though this insertion did not affect the repression of *BrFLC5* expression following prolonged cold treatments. High *BrFLC5* expression levels were found to be linked to this insertion using an F_2_ population derived from crossed accessions having homozygous type B and type C *BrFLC5* alleles. Though the possibility that the other sequence variations of *BrFLC5* between parental lines of the F_2_ population results in the difference of *BrFLC5* expression was not completely excluded, we suggest that this insertion leads to increased *BrFLC5* expression in pre-vernalized plants. However, it is necessary to test for association using more accessions, to test for association using segregating populations, or to prove it by promoter assay.

Leafy and root *B. rapa* vegetables need a high requirement for vernalization to prevent premature bolting, which reduces the yield and quality of products (Akter et al. 2021). A higher expression level before vernalization is one important characteristic of a high vernalization requirement (Takada et al. 2019; Akter et al. 2021); thus, the higher functional *BrFLC5* expression level in pre-vernalized material like type C *BrFLC5* allele could be important for a high vernalization requirement. A variation of *BrFLC5* expression levels among accessions having functional *BrFLC5* alleles, type B and type C, in pre-vernalized plants was observed in this study. Thus, if there is an association between this insertion and increased *BrFLC5* expression before vernalization, developing a DNA marker identifying this insertion will be useful for marker-assisted selection.

## Conclusion

Some accessions have a functional *BrFLC5* allele, and this functional difference between varieties may be associated with differences in vernalization requirement in *B. rapa*. There is a variation of *BrFLC5* expression levels between accessions prior to vernalization. Though the effect may not be significant due to *BrFLC5* having the lowest expression levels among the four *FLC* paralogs, higher functional *BrFLC5* expression might contribute to increased vernalization requirement. Thus, the type C allele with a functional *BrFLC5* and higher *BrFLC5* expression levels may be useful for developing high bolting-resistant breeding in *B. rapa* vegetables.

## Supplementary information

ESM 1 (PPTX 2053 kb)

ESM 2 (XLSX 21 kb)

## Data Availability

DDBJ; accession no. DRA015525.
