# Supplementary material for: Characterization of FLOWERING LOCUS C 5 in Brassica rapa L
Source: Mol Breed. 2023 Jul 19;43(8):58. doi: 10.1007/s11032-023-01405-0 (PMC10356691; doi:10.1007/s11032-023-01405-0)
Supplement: Supplementary file 1 — (PPTX 2053 kb) [file 11032_2023_1405_MOESM1_ESM.pptx]

## Slide 1
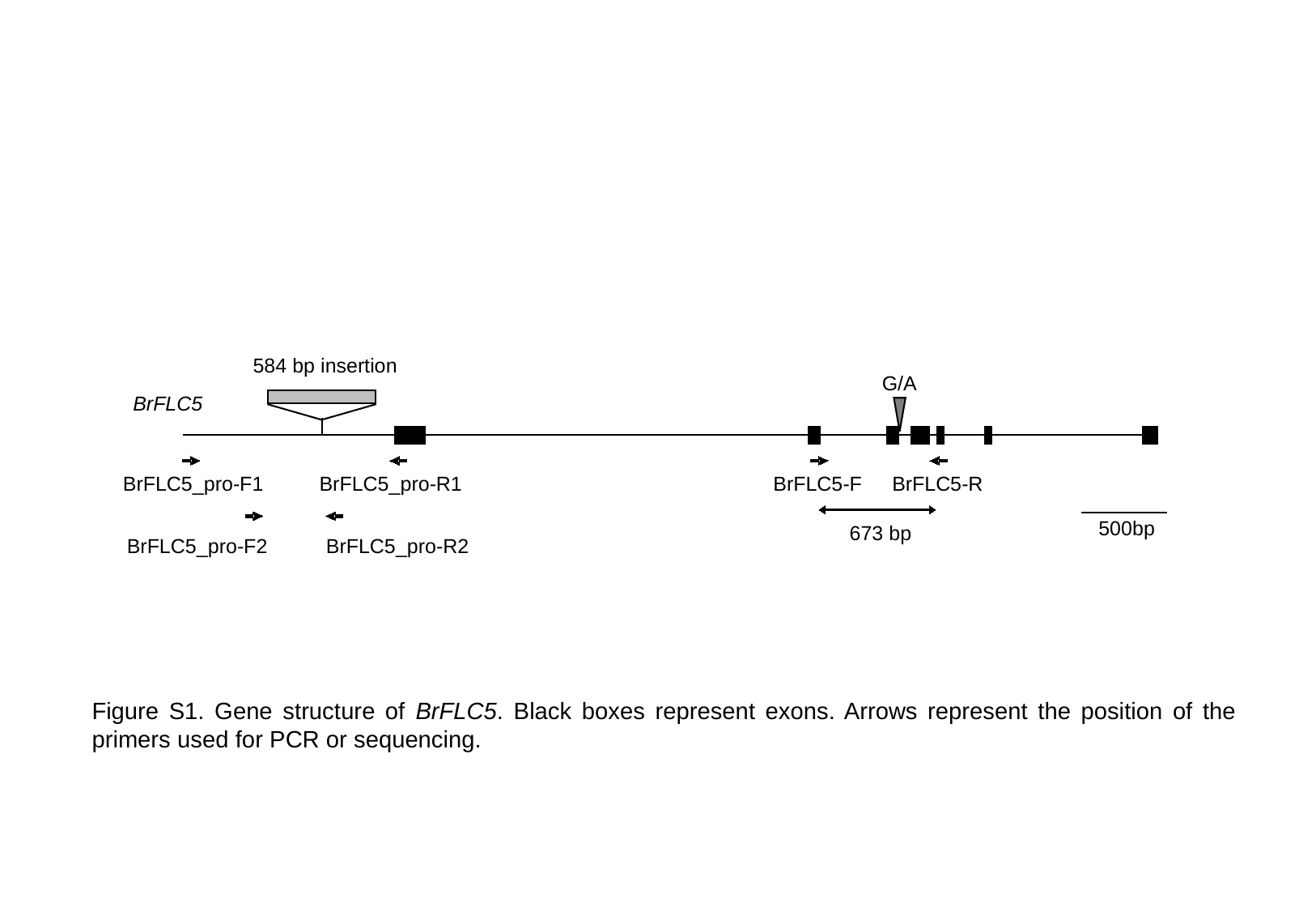

584 bp insertion
G/A
BrFLC5
BrFLC5_pro-F1
BrFLC5_pro-R1
BrFLC5-F
BrFLC5-R
500bp
673 bp
BrFLC5_pro-F2
BrFLC5_pro-R2
Figure S1. Gene structure of BrFLC5. Black boxes represent exons. Arrows represent the position of the primers used for PCR or sequencing.

## Slide 2
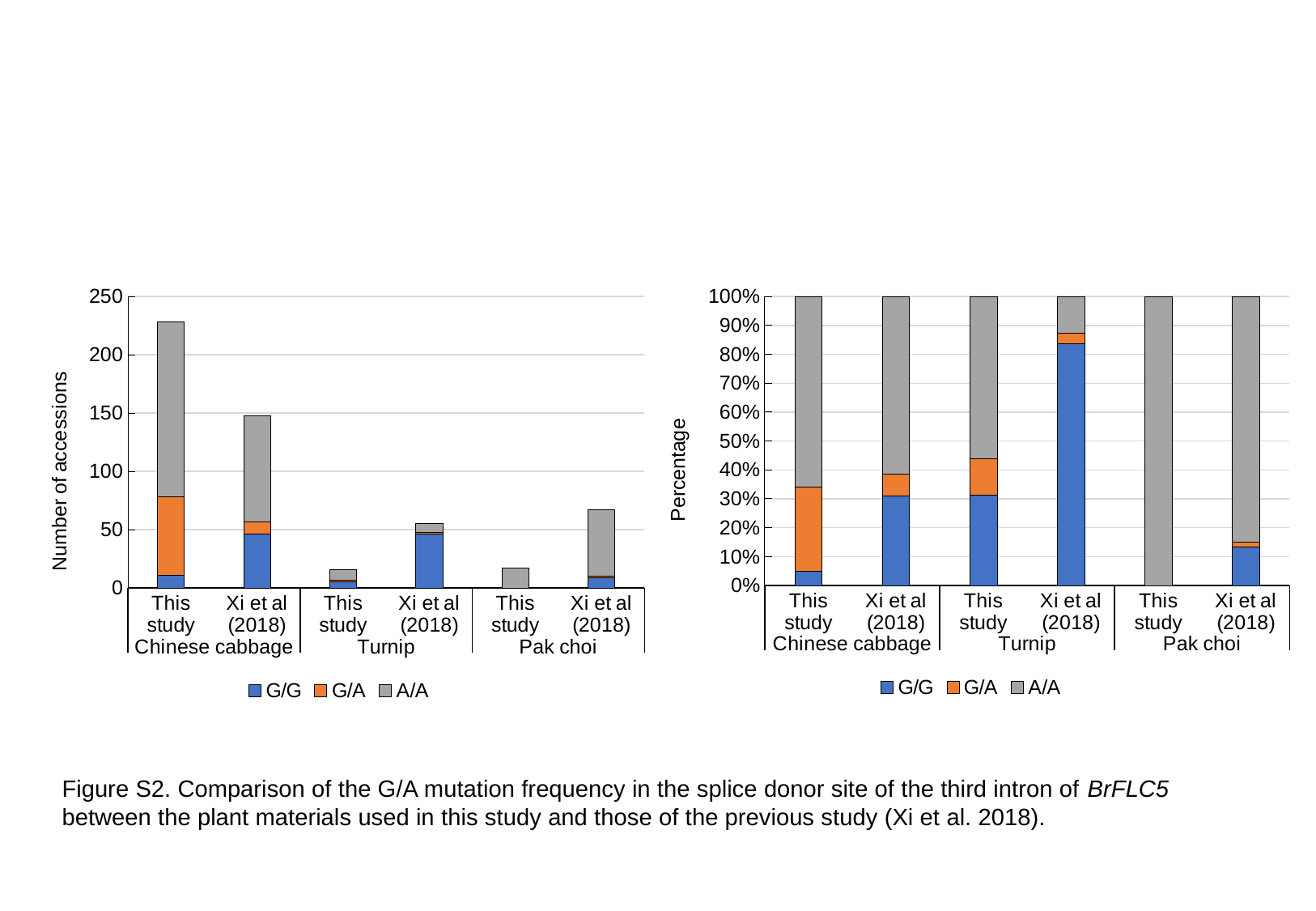

### Chart
| Category | G/G | G/A | A/A |
|---|---|---|---|
| This study | 11.0 | 67.0 | 150.0 |
| Xi et al (2018) | 46.0 | 11.0 | 91.0 |
| This study | 5.0 | 2.0 | 9.0 |
| Xi et al (2018) | 46.0 | 2.0 | 7.0 |
| This study | 0.0 | 0.0 | 17.0 |
| Xi et al (2018) | 9.0 | 1.0 | 57.0 |
### Chart
| Category | G/G | G/A | A/A |
|---|---|---|---|
| This study | 11.0 | 67.0 | 150.0 |
| Xi et al (2018) | 46.0 | 11.0 | 91.0 |
| This study | 5.0 | 2.0 | 9.0 |
| Xi et al (2018) | 46.0 | 2.0 | 7.0 |
| This study | 0.0 | 0.0 | 17.0 |
| Xi et al (2018) | 9.0 | 1.0 | 57.0 |Figure S2. Comparison of the G/A mutation frequency in the splice donor site of the third intron of BrFLC5 between the plant materials used in this study and those of the previous study (Xi et al. 2018).

## Slide 3
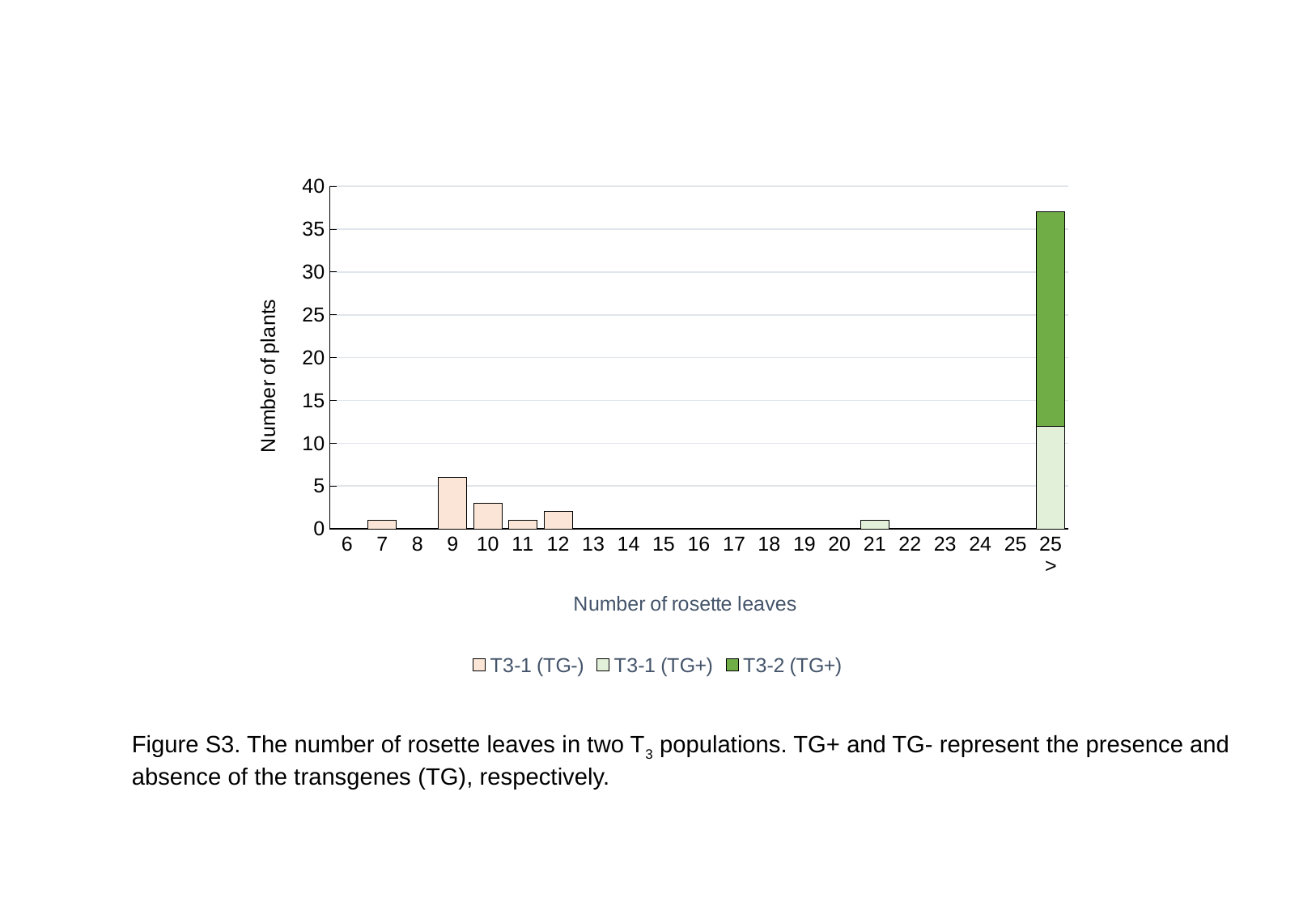

### Chart
| Category | T3-1 (TG-) | T3-1 (TG+) | T3-2 (TG+) |
|---|---|---|---|
| 6 | None | None | None |
| 7 | 1.0 | None | None |
| 8 | None | None | None |
| 9 | 6.0 | None | None |
| 10 | 3.0 | None | None |
| 11 | 1.0 | None | None |
| 12 | 2.0 | None | None |
| 13 | None | None | None |
| 14 | None | None | None |
| 15 | None | None | None |
| 16 | None | None | None |
| 17 | None | None | None |
| 18 | None | None | None |
| 19 | None | None | None |
| 20 | None | None | None |
| 21 | None | 1.0 | None |
| 22 | None | None | None |
| 23 | None | None | None |
| 24 | None | None | None |
| 25 | None | None | None |
| 25> | None | 12.0 | 25.0 |Figure S3. The number of rosette leaves in two T3 populations. TG+ and TG- represent the presence and absence of the transgenes (TG), respectively.

## Slide 4
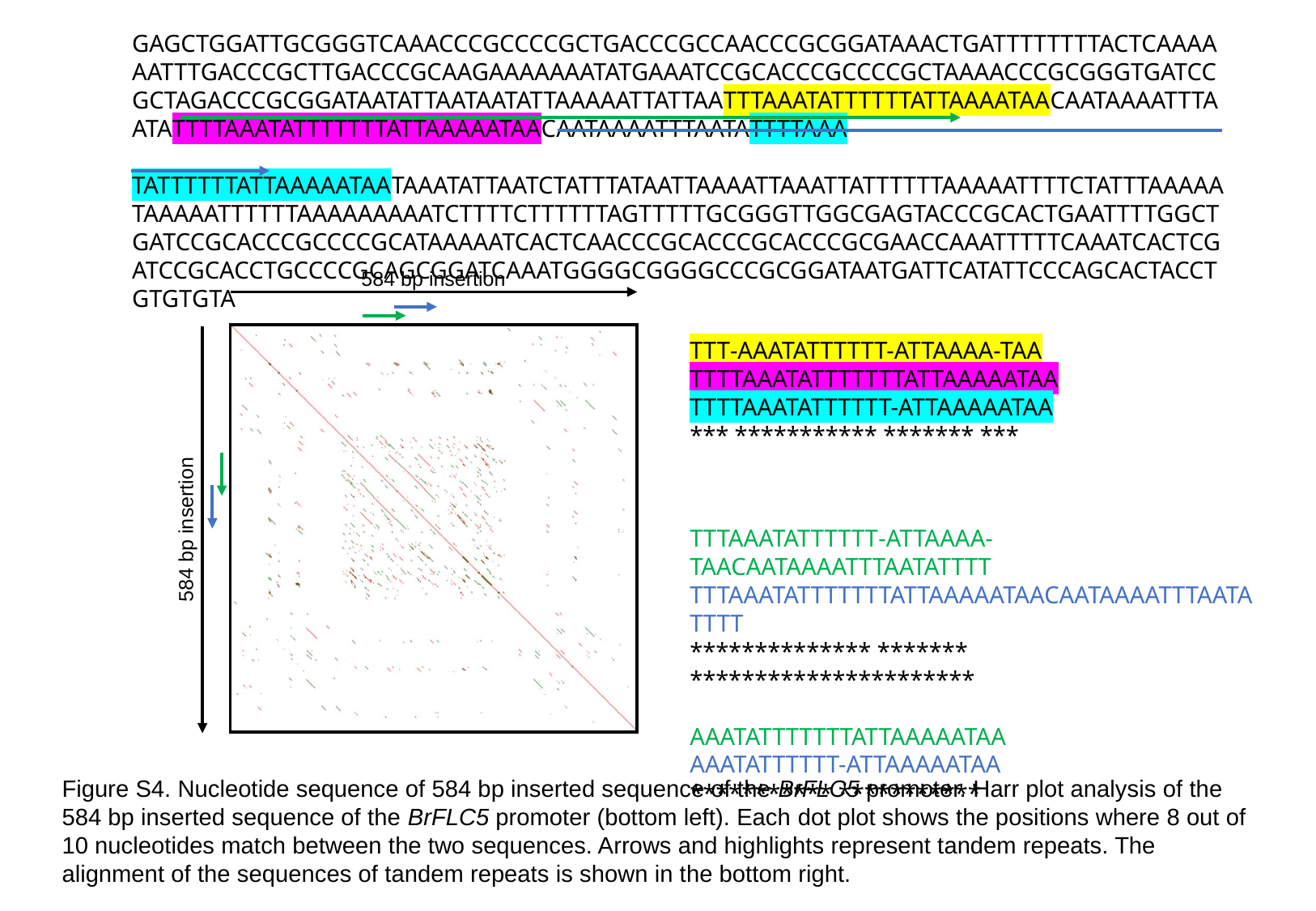

GAGCTGGATTGCGGGTCAAACCCGCCCCGCTGACCCGCCAACCCGCGGATAAACTGATTTTTTTTACTCAAAAAATTTGACCCGCTTGACCCGCAAGAAAAAAATATGAAATCCGCACCCGCCCCGCTAAAACCCGCGGGTGATCCGCTAGACCCGCGGATAATATTAATAATATTAAAAATTATTAATTTAAATATTTTTTATTAAAATAACAATAAAATTTAATATTTTAAATATTTTTTTATTAAAAATAACAATAAAATTTAATATTTTAAA
TATTTTTTATTAAAAATAATAAATATTAATCTATTTATAATTAAAATTAAATTATTTTTTAAAAATTTTCTATTTAAAAATAAAAATTTTTTAAAAAAAAATCTTTTCTTTTTTAGTTTTTGCGGGTTGGCGAGTACCCGCACTGAATTTTGGCTGATCCGCACCCGCCCCGCATAAAAATCACTCAACCCGCACCCGCACCCGCGAACCAAATTTTTCAAATCACTCGATCCGCACCTGCCCCGCAGCGGATCAAATGGGGCGGGGCCCGCGGATAATGATTCATATTCCCAGCACTACCTGTGTGTA
584 bp insertion
584 bp insertion
TTT-AAATATTTTTT-ATTAAAA-TAA
TTTTAAATATTTTTTTATTAAAAATAA
TTTTAAATATTTTTT-ATTAAAAATAA
*** *********** ******* ***
TTTAAATATTTTTT-ATTAAAA-TAACAATAAAATTTAATATTTT
TTTAAATATTTTTTTATTAAAAATAACAATAAAATTTAATATTTT
************** ******* **********************
AAATATTTTTTTATTAAAAATAA
AAATATTTTTT-ATTAAAAATAA
*********** ***********
Figure S4. Nucleotide sequence of 584 bp inserted sequence of the BrFLC5 promoter. Harr plot analysis of the 584 bp inserted sequence of the BrFLC5 promoter (bottom left). Each dot plot shows the positions where 8 out of 10 nucleotides match between the two sequences. Arrows and highlights represent tandem repeats. The alignment of the sequences of tandem repeats is shown in the bottom right.

## Slide 5
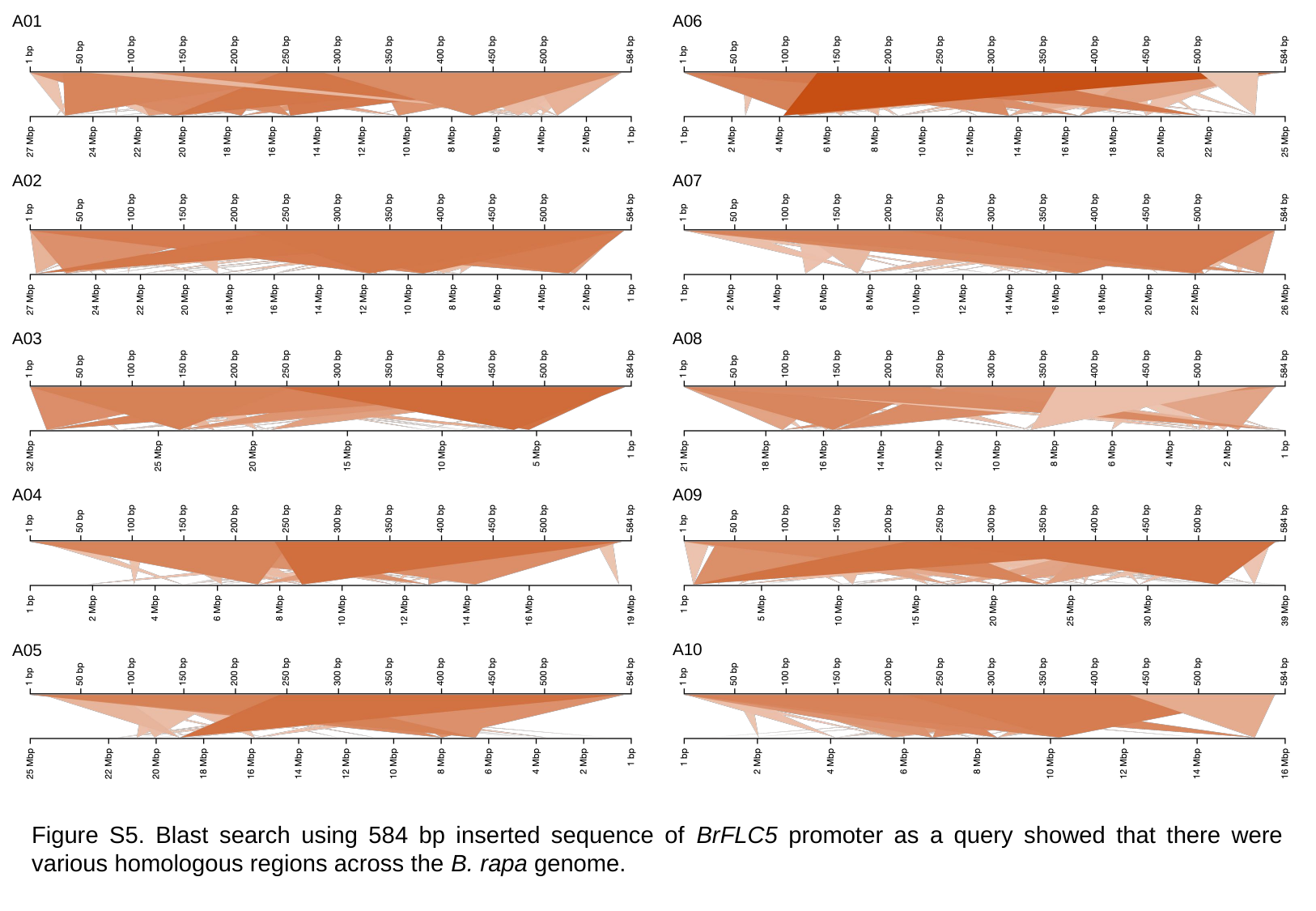

A01
A06
A02
A07
A03
A08
A04
A09
A10
A05
Figure S5. Blast search using 584 bp inserted sequence of BrFLC5 promoter as a query showed that there were various homologous regions across the B. rapa genome.

## Slide 6
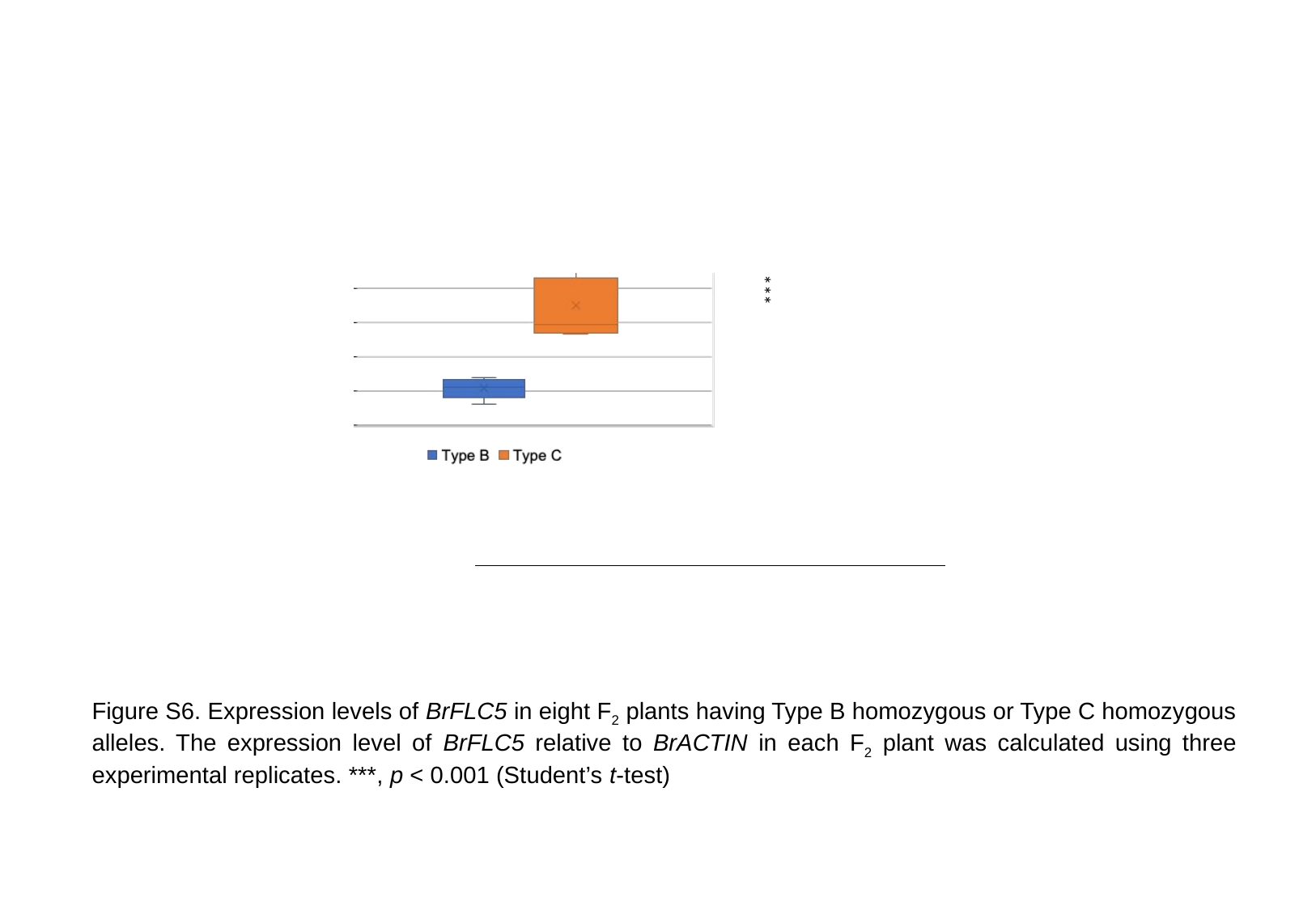

***
Figure S6. Expression levels of BrFLC5 in eight F2 plants having Type B homozygous or Type C homozygous alleles. The expression level of BrFLC5 relative to BrACTIN in each F2 plant was calculated using three experimental replicates. ***, p < 0.001 (Student’s t-test)

## Slide 7
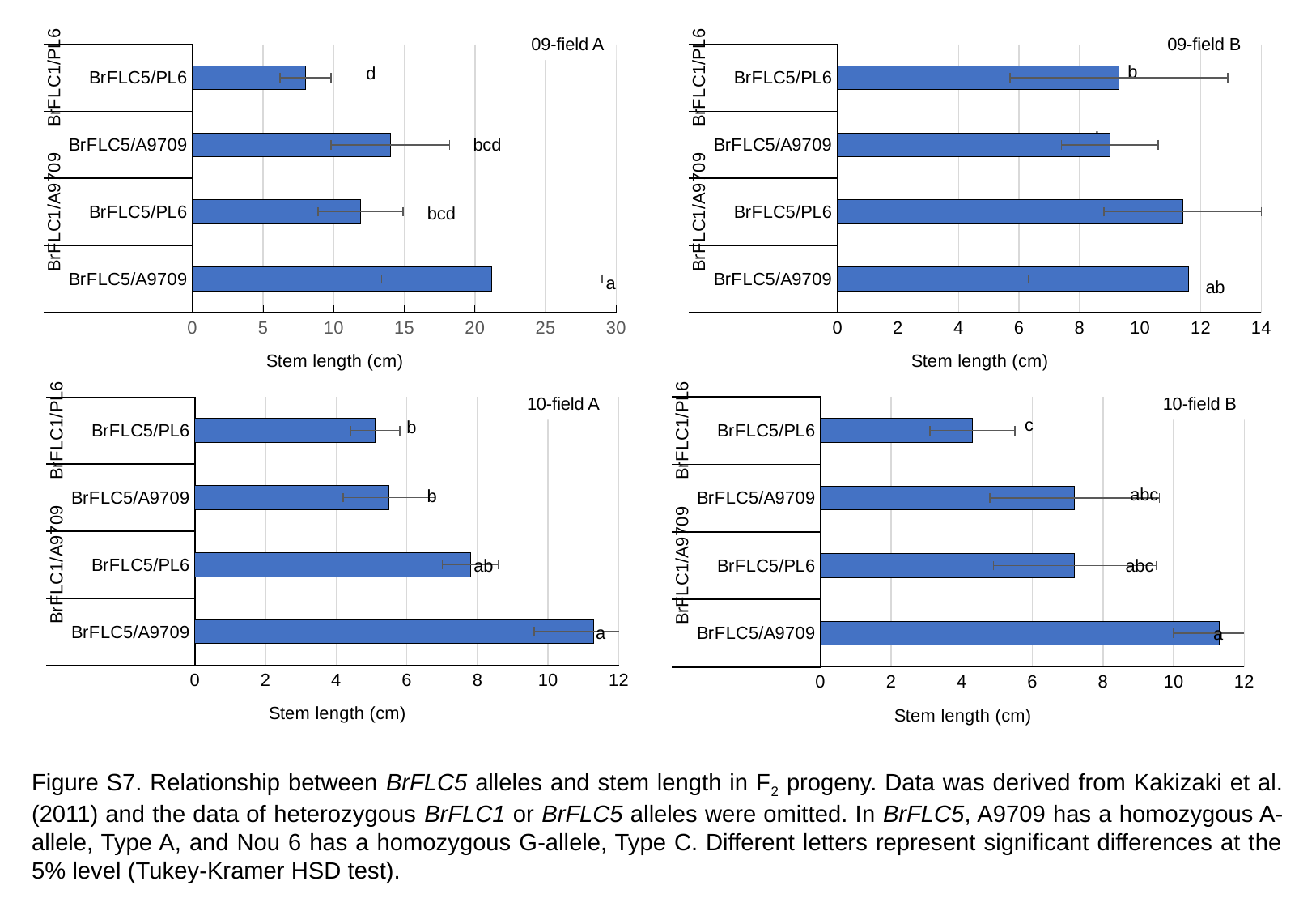

### Chart
| Category | |
|---|---|
| BrFLC5/A9709 | 11.6 |
| BrFLC5/PL6 | 11.4 |
| BrFLC5/A9709 | 9.0 |
| BrFLC5/PL6 | 9.3 |
### Chart
| Category | |
|---|---|
| BrFLC5/A9709 | 21.2 |
| BrFLC5/PL6 | 11.9 |
| BrFLC5/A9709 | 14.0 |
| BrFLC5/PL6 | 8.0 |09-field A
09-field B
b
d
ab
bcd
ab
bcd
a
ab
### Chart
| Category | |
|---|---|
| BrFLC5/A9709 | 11.3 |
| BrFLC5/PL6 | 7.8 |
| BrFLC5/A9709 | 5.5 |
| BrFLC5/PL6 | 5.1 |
### Chart
| Category | |
|---|---|
| BrFLC5/A9709 | 11.3 |
| BrFLC5/PL6 | 7.2 |
| BrFLC5/A9709 | 7.2 |
| BrFLC5/PL6 | 4.3 |10-field A
10-field B
c
b
abc
b
ab
abc
a
a
Figure S7. Relationship between BrFLC5 alleles and stem length in F2 progeny. Data was derived from Kakizaki et al. (2011) and the data of heterozygous BrFLC1 or BrFLC5 alleles were omitted. In BrFLC5, A9709 has a homozygous A-allele, Type A, and Nou 6 has a homozygous G-allele, Type C. Different letters represent significant differences at the 5% level (Tukey-Kramer HSD test).
